# Reconstruction of the interosseous talocalcaneal ligament using allograft for subtalar joint stabilization is effective

**DOI:** 10.1007/s00167-023-07622-6

**Published:** 2023-11-13

**Authors:** Elvira Iglesias-Durán, Francisco Guerra-Pinto, Cristina Ojeda-Thies, Jesús Vilá-Rico

**Affiliations:** 1 Hospital Monográfico ASEPEYO, Calle Joaquín de Cardenas 2, Coslada, Madrid, Spain; 2 Universidad Europea de Madrid, Madrid, Spain; 3 Hospital Quirónsalud Ruber, Madrid, Spain; 4 Hospital Ortopédico de Sant’Ana, Parede, Portugal; 5 Hospital da Cruz Vermelha Portuguesa, Lisbon, Portugal; 6 Hospital da Luz Oeiras, Oeiras, Portugal; 7 NOVA Medical School, Lisbon, Portugal; 8 Hospital Universitario 12 de Octubre, Madrid, Spain; 9 Universidad Complutense de Madrid, Madrid, Spain

**Keywords:** Interosseous talocalcaneal ligament, subtalar joint instability, Graft reconstruction, Biomechanical study, Hindfoot instability

## Abstract

**Purpose:**

The aim of this study was to assess the biomechanical effects of subtalar ligament injury and reconstruction on stability of the subtalar joint in all three spatial planes.

**Methods:**

Fifteen fresh frozen cadaveric legs were used, with transfixed tibiotalar joints to isolate motion to the subtalar joint. An arthrometer fixed to the lateral aspect of the calcaneus measured angular displacement in all three spatial planes on the inversion and eversion stress tests. Stress manoeuvres were tested with the intact joint, and then repeated after sequentially sectioning the inferior extensor retinaculum (IER), cervical ligament (CL), interosseous talocalcaneal ligament (ITCL), arthroscopic graft reconstruction of the ITCL, and sectioning of the calcaneo-fibular ligament (CFL).

**Results:**

Sectioning the ITCL significantly increased angular displacement upon inversion and eversion in the coronal and sagittal planes. Reconstruction of the ITCL significantly improved angular stability against eversion in the axial and sagittal planes, and against inversion in the axial and coronal planes, at the zero time point after reconstruction. After sectioning the CFL, resistance to eversion decreased significantly in all three planes.

**Conclusion:**

Progressive injury of ligamentous stabilisers, particularly the ITCL, led to increasing angular displacement of the subtalar joint measured with the inversion and eversion stress tests, used in clinical practice. Reconstruction of the ITCL using tendon graft significantly stabilised the subtalar joint in the axial and sagittal planes against eversion and in the axial and coronal planes against inversion, immediately after surgery.

## Introduction

**Fig. 1:**
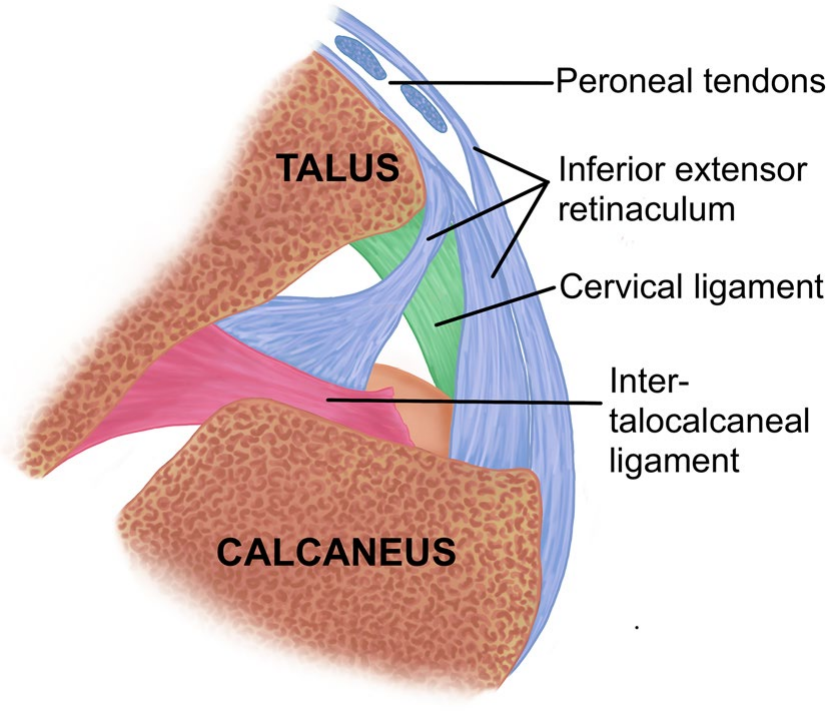
Intrinsic ligamentous stabilisers of the subtalar joint (coronal section): Inferior extensor retinaculum (blue), cervical ligament (green), inter-talocalcaneal ligament (red)

The subtalar joint enables translation in all three planes between the foot and the rest of the lower limb, and is key for adequate hindfoot mechanics during the loading response of the hindfoot between the initial heel contact and the mid-stance phases of the gait cycle. Its stability resides in the configuration of the anterior and posterior subtalar joint surfaces as well as several ligamentous structures (Fig. 1) [6, 25, 27]: the inferior extensor retinaculum (IER), the calcaneo-fibular ligament (CFL), the cervical ligament (CL) and finally the inter-talocalcaneal ligament (ITCL), which comprises an anterior and a posterior fascicle that rotate over each other regulating subtalar motion—similar to the cruciate ligaments of the knee [5, 6, 8, 10, 18, 19, 25, 39, 40].

Subtalar joint instability is an entity commonly neglected within the scope of lateral ankle instability in patients of all ages; up to 25% of chronic ankle instabilities have associated subtalar instability [2, 24, 30], which could account for some of the cases with persistent symptoms after isolated repair or reconstruction of the anterior talo-fibular ligament [38]. A recent study found 90% of patients with chronic lateral ankle instability and sinus tarsi pain had ITCL tears [36]. Patients complain of giving way of the ankle, especially over irregular terrain or during athletic activity, and report pain on the lateral side of the hindfoot and the sinus tarsi. Symptoms are unspecific and similar to chronic lateral ankle instability and are difficult to assess, even for experienced clinicians [1, 23, 37].

Controversy exists surrounding the main structures injured that contribute to subtalar instability; some authors consider the ITCL and CL the main stabilisers of the joint, while others defend the CFL ligament’s role [12, 13, 18, 20, 22, 30–32, 34]. Treatment is similar to that of chronic lateral ankle instability, ranging from conservative to surgical, with non-anatomic and anatomic reconstruction techniques [13]. Most experience is limited to retrospective case series [32], with only few prospective studies [26]. An arthroscopic technique for ITCL reconstruction has recently been described [9]. Ankle and subtalar arthroscopy are a useful aid in these cases, as it allows for assessment of intra-articular lesions, confirmation of ligamentous injuries and facilitates the ideal placement of bone tunnels to ensure an anatomically optimal reconstruction.

The subtalar joint is hypothesised by the authors of this study to be stabilised by allograft reconstruction of the ITCL, under cadaver-simulated conditions of subtalar instability. Determining the contribution of the IER, CL, ITCL and CFL on subtalar joint stability in all three spatial planes, as well as the effect of ITCL allograft reconstruction on angular stability of the subtalar joint in a cadaveric model, was a secondary goal.

## Materials and methods

### Specimens

Fifteen fresh frozen cadaveric ankles without deformities, morphologic alterations or scars were used. The study was performed at the Department of Anatomy of the Francisco de Vitoria University in Madrid, Spain, and fulfilled local legal and ethical criteria for cadaveric studies (study number 001/2018). The corpses proceed from the University’s Body Donation Program that had been stored at – 15 °C and slowly thawed for at least 24 h prior to the study to avoid stiffness that would interfere with measurements. Each specimen was sectioned below the knee joint maintaining at least 20 cm of tibia and fibula.

### Angular measurements

**Fig. 2:**
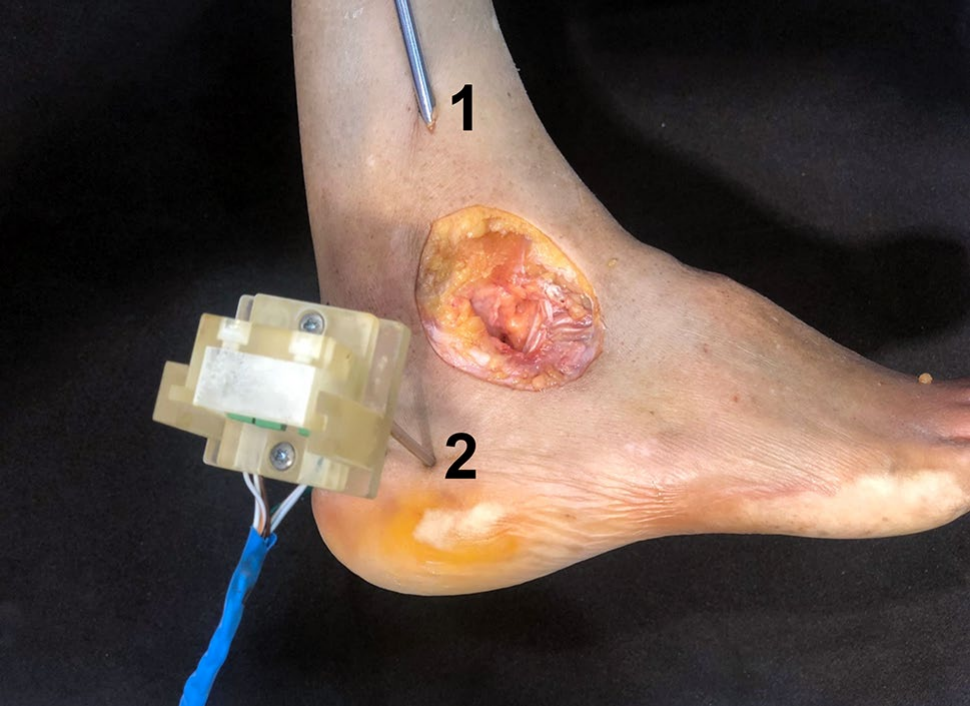
Experimental setup. The tibiotalar joint is blocked using two 3.0 mm pins across the joint (1), leaving only the calcaneus and midfoot free. The arthrometer is fixed to the lateral aspect of the calcaneus using two Kirschner wires (2)

An arthrometer specifically designed to measure angular displacements in all three anatomic planes (axial, coronal, sagittal) [6] was used. A sensor to the calcaneus (Fig. 2) through two 3 mm Kirschner wires drilled perpendicularly to the limb axis into the lateral aspect of the calcaneus at the level of the fibula, parallel to the longitudinal axis of the calcaneus was attached. Only the calcaneus was free to move, with the tibiotalar joint fixed by crossed Steinmann pins. The sensor was a Mpu-6050 inertial measurement unit that has a triaxial accelerometer and a triaxial gyroscope, with six degrees of freedom, connected to an Arduino Mega 2560 computer. The arthrometer’s software gives the angular values of the difference between the sensor’s initial orientation and the final orientation after performing the stability manoeuvres made it possible to analyse angular displacement of the calcaneus in all three planes simultaneously, using Tait–Bryan angles, to three decimal points of a degree.

In the axial plane, external rotation was assigned positive values (*x* > 0), while negative values (*x* < 0) corresponded to internal rotation. In the coronal plane, inversion received positive values, while eversion was measured in negative values. In the sagittal plane, positive values corresponded to plantarflexion and negative values to dorsiflexion.

The stability manoeuvres performed were the inversion stress test (IST) and eversion stress test (EST) [16]. In the inversion stress test, the examiner holds the calcaneus with the thumb on the lateral side and applies forced varus (inversion) while holding the tibia with the contralateral hand. In the eversion stress test, the examiner holds the limb the same fashion but applies forced valgus (eversion). The forces were applied manually and by the lead investigator in the same order. Each manoeuvre was repeated three times, and the average of the three measurements was used. The intra-rater reliability correlation coefficients (ICC) between measures were 0.83 (0.65–0.89) for the inversion tests and 0.79 (0.63–0.87) for the eversion tests. Bending of the wires or displacement of the sensor was not observed and would have led to recalibration of the specimen or loss of the sample, depending on the stage of the experiment.

### Experimental protocol

After transfixing the tibiotalar joint with Steinmann pins to block all motion at this level and isolate angular displacements of the hindfoot to the subtalar joint, the specimens were mounted and the arthrometer sensor was fixed with pins to the lateral aspect of the calcaneus (Fig. 2); subtalar angular displacement was measured in each specimen. A 4.5–5.0 graft is required for ITCL reconstruction, so an extensor hallucis longus tendon graft was then obtained from each specimen at this stage, for ligament reconstruction further on. The subtalar joint was not affected by graft harvest.

**Fig. 3:**
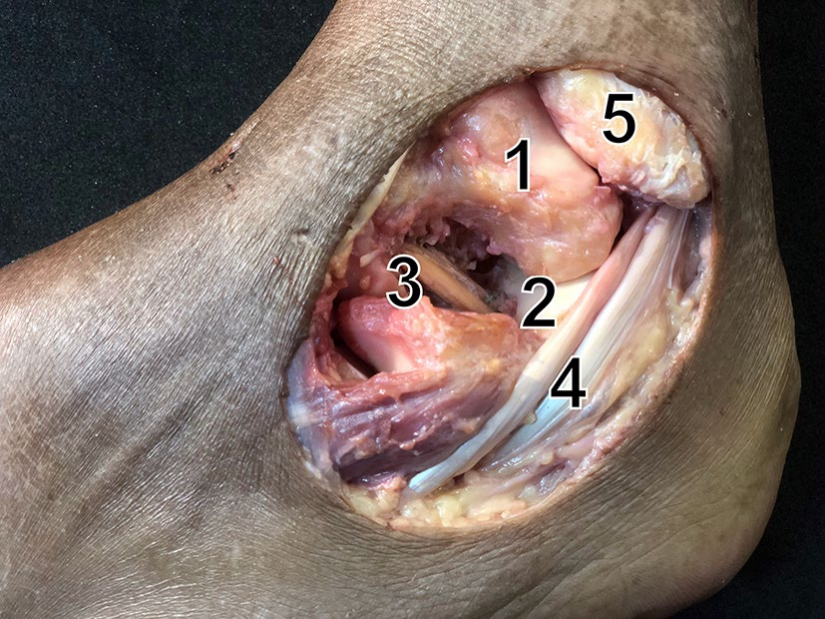
Inter-talocalcaneal ligament graft reconstruction. (1) Talus; (2) Calcaneus; (3) Graft reconstruction; (4) Peroneal ligaments; (5) Fibula

- 1. Initially, with the subtalar joint intact, the following manoeuvres were performed: inversion (I) and eversion (E), plantar flexion (PF) and dorsiflexion (DF), internal rotation (IR) and external rotation (ER), and the angular displacement detected by the arthrometer in the three anatomical planes was recorded.
- 2. Next, the IER was sectioned and the stability examination manoeuvres were repeated.
- 3. Next, the CL was sectioned and the stability examination manoeuvres were repeated.
- 4. The ITCL was then sectioned and the stability examination manoeuvres were performed.
- 5. Subsequently, anatomical reconstruction using a graft from the same cadaver was performed. The ITCL was then anatomically reconstructed with a bifascicular technique using the harvested extensor hallucis longus graft, with a complete calcaneal tunnel and a talar half tunnel, and fixed using the dynamic ACL TightRope^®^ system (Arthrex, Naples, FL, USA) at the talar end and a 6.25 mm biotenodesis screw (Arthrex, Naples, FL, USA) at the calcaneal end, with the subtalar joint in slight eversion and dorsiflexion (Fig. 3) [6]. Angular stability was assessed again after reconstruction and the angular movements were recorded.
- 6. Finally, the CFL was sectioned and the examination manoeuvres were performed in order to measure stability using the arthrometer. This process was then repeated after sectioning the reconstructed ITCL.

### Statistical analysis

The number of specimens was calculated by applying the formula established for infinite populations and rounding its results to the highest integer, using previously published information regarding the distribution of measurements in the general population (population variance: 1.15) and the margin of error of the measurement device [27, 41], with a clinically significant threshold at 20% difference between groups. Correction for dropouts was considered unnecessary due to the study characteristics, and the estimated sample size was 15.

Qualitative variables are presented as frequencies and percentage values, while quantitative variables are summarised as median and interquartile ranges because the sample was too small (Table 1). Statistical inference was performed for the inversion and eversion stress tests using Wilcoxon’s signed rank test. Significance was set at *p* < 0.05. Data was analysed using SPSS 21.0^®^ for Windows (IBM Corp, Armonk, NY, USA).

**Table 1:** Different measurements in the axial, coronal and sagittal planes

		Axial plane		Coronal		Sagittal	
Joint status	Comparison	Degrees (median, IQR)	*p* value	Degrees (median, IQR)	*p* value	Degrees (median, IQR)	*p* value
*Eversion test, angular displacement*							
(1) Intact subtalar joint	–	− 4.8 (− 8.9 to − 2.4)	n/a	− 8.5 (− 12.4 to − 4.1)	n/a	− 5.6 (− 8.4 to − 2.0)	n/a
(2) Sectioned retinaculum	(1) vs. (2)	− 7.0 (− 11.1 to − 3.1)	n.s	− 8.8 (− 13.0 to − 3.9)	n.s	− 6.0 (− 9.4 to − 3.2)	n.s
(3) Sectioned CL	(1) vs. (3)	− 9.0 (− 12.5 to − 3.8)	0.001*	− 8.8 (− 14.0 to − 4.4)	n.s	− 8.0 (− 10.4 to − 4.7)	0.001*
(4) Sectioned ITCL	(1) vs. (3) + (4)	− 9.5 (− 12.6 to − 5.6)	n.s	− 10.9 (− 14.9 to − 5.8)	0.002*	− 10.9 (− 14.9 to − 5.8)	0.001*
(5) Ligament reconstruction	(5) vs. (3) + (4)	− 7.2 (− 9.3 to − 5.3)	0.033*	− 7.9 (− 12.3 to − 6.1)	n.s	− 8.6 (− 10.7 to − 8.1)	0.015*
(6) Ligament reconstruction with sectioned CFL	(5) vs. (6)	− 9.8 (− 14.9 to − 6.2)	0.008*	− 11.0 (− 14.1 to − 8.1)	0.003*	− 8.8 (− 10.9 to − 5.4)	0.021*
(7) Sectioned CL + ITCL + CFL	(5) vs. (7)	− 10.3 (− 15.4 to − 6.1)	0.003*	− 12.9 (− 15.8 to − 9.1)	0.001*	− 10.5 (− 13.6 to − 8.5)	0.001*
*Inversion test, angular displacement*							
(1) Intact subtalar joint	–	1.5 (0.9–4.0)	n/a	4.4 (3.5–5.4)	n/a	0.3 (0.0–1.4)	n/a
(2) Sectioned retinaculum	(1) vs. (2)	2.3 (1.2–3.7)	n.s	4.3 (2.6 –5.7)	n.s	0.8 (0.2–1.6)	n.s
(3) Sectioned CL	(1) vs. (3)	1.7 (0.8–3.7)	n.s	5.3 (3.4–6.2)	n.s	0.7 (0.3–1.8)	n.s
(4) Sectioned ITCL	(1) vs. (3) + (4)	2.6 (0.5–5.3)	n.s	5.8 (4.3–7.0)	0.016*	1.5 (0.4–3.2)	0.017*
(5) Ligament reconstruction	(5) vs. (3) + (4)	0.5 (0.1–1.9)	0.031*	3.4 (2.7–4.3)	0.002*	0.6 (− 0.1 to 1.8)	n.s
(6) Ligament reconstruction with sectioned CFL	(5) vs. (6)	0.4 (− 0.3 to 1.7)	n.s	6.0 (5.2–7.1)	0.001*	0.8 (− 0.2 to 1.3)	n.s
(7) Sectioned CL + ITCL + CFL	(5) vs. (7)	2.4 (0.9–5.2)	n.s	7.8 (5.2–10.1)	0.001*	1.3 (− 0.1 to 3.3)	n.s

## Results

For the IST, sectioning the ITCL added to laxity in the coronal and sagittal planes, and ligament reconstruction returned stability to practically normal values. When evaluating varus displacement in the coronal plane, sectioning the ITCL led to increasing instability. Sectioning the CFL after ITCL reconstruction did not appear to have any significant effect. Ligament reconstruction returned stability in the coronal plane, while sectioning the CFL significantly affected stability against varus. In the sagittal plane, sectioning the retinaculum affected stability against plantarflexion more than sectioning the cervical ligament; injury to the ITCL had a significant effect on joint laxity that was remedied by ligament reconstruction.

For the EST, when assessing rotation in the axial plane, sectioning the CL significantly increased external rotation, but adding injury of the ITCL did not add to rotational instability. Laxity restoration against external rotation improved significantly when reconstructing the ligament and deteriorated when adding injury to the CFL. Upon examination of valgus displacement in the coronal plane, neither injuring the retinaculum nor the CL had an effect on valgus laxity of the subtalar joint, but sectioning the ITCL did show a significant effect. Ligament reconstruction showed values similar to the sectioned IER, but adding CFL injury added to valgus laxity in the coronal plane. In the sagittal plane, injuring structures sequentially led to increasing instability in dorsiflexion. Ligament reconstruction returned laxity to nearly normal values. Sectioning the CFL affected laxity in dorsiflexion.

Reconstruction of the ITCL significantly improved angular stability of the subtalar joint against eversion in the axial, and sagittal planes, and against inversion in the axial and coronal planes, at the zero time point after reconstruction.

**Fig. 4:**
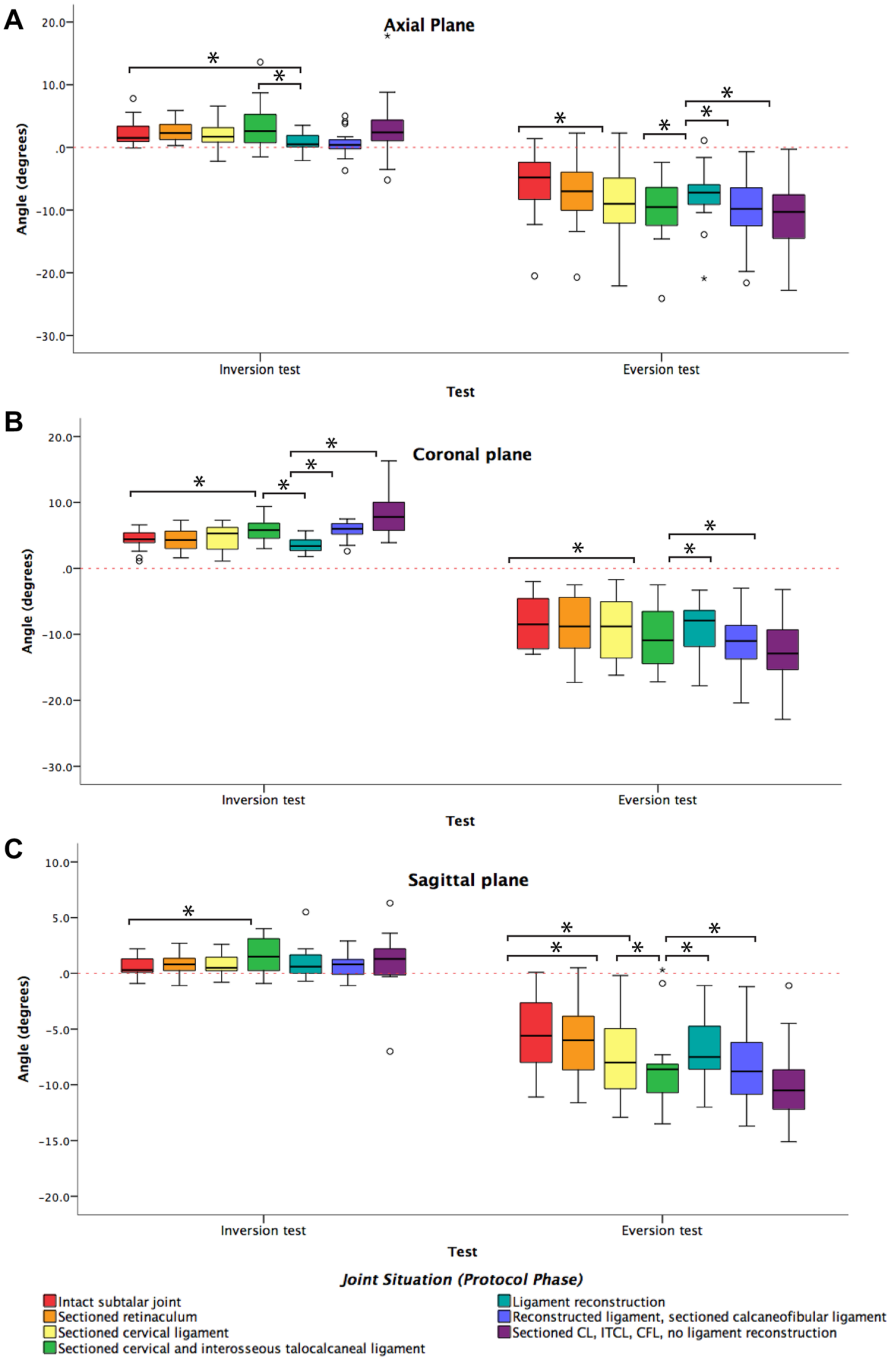
Angular displacements measured in the axial (**A**), coronal (**B**) and sagittal (**C**) planes with the inversion and eversion tests, for each stage of the experimental protocol. Significant comparisons are marked by bars and asterisks

In the comparative study of the intact subtalar joint and the reconstruction, no statistically significant differences were found, except in the axial plane with the inversion manoeuvre (p = 0.001), which can be interpreted as the reconstruction stabilising the subtalar joint in the inversion manoeuvres in the coronal (n.s.) and sagittal (n.s.) plane and in the eversion manoeuvre in the axial (n.s.), coronal (n.s.) and sagittal (n.s.) planes (Fig. 4).

## Discussion

The most important finding of this study was the biomechanical contribution of the different ligaments around the subtalar joint on angular displacement in all three anatomical planes and the effect of graft reconstruction of the interosseous talocalcaneal ligament. Injury to the ITCL added to laxity in the coronal and sagittal planes upon inversion, and ligament reconstruction returned stability to practically normal values. Sectioning the CFL after ITCL reconstruction did not appear to have any significant effect against inversion, but increased laxity in external rotation upon eversion.

Recent studies were unable to identify a specific ligament that acted as the main stabiliser of the subtalar joint, and suggested that the ligaments act in conjunction against a combination of movements, stabilising the joint in all three planes [24, 28]. Eversion of the tarsus combines dorsiflexion, pronation and abduction, while inversion includes plantarflexion, supination and adduction of the foot; our study design, concurrently analysing all six degrees of freedom, is especially suited for addressing this complex joint. To avoid misinterpretations of terms like supination and pronation, we preferred to follow the example of authors like Sangerorzan et al. and Guerra-Pinto et al., and referred to displacement in three orthogonal spatial planes (axial, coronal, sagittal) [7, 35].

The IER was observed to restrict subtalar movement in the axial plane (internal / external rotation) in inversion and to a lesser degree in eversion. The CL ligament stabilised against external rotation in eversion, in agreement with Kjærsgaard-Andersen et al. [18], but not in inversion, as suggested by other authors [21, 33]. Finally, the ITCL was found to be an important stabiliser in the coronal and sagittal planes in our study, in both inversion and eversion. Injury to the calcaneo-fibular ligament significantly led to instability of the subtalar joint in all three spatial planes against eversion, and in the coronal plane (varus) against inversion. Other authors demonstrated that sectioning the lateral ankle stabilisers including the CL did not lead to significant changes of subtalar joint kinematics during weight-bearing (closed chain) movements [29]. Most studies analysing ligament instability used models in non-weight-bearing (open chain) scenarios, as in the present study [4, 24, 30, 34, 41] and a recent cadaveric study found that the detection of subtalar joint instability was attenuated under conditions of simulated weight-bearing [3]. Whether the ITFL or the CFL has more influence of subtalar joint instability is a topic of discussion. An angle of the CFL relative to the anterior talo-fibular ligament of less than 70º has recently been found to be highly suggestive of subtalar joint instability [17]. Unfortunately, which ligament influenced subtalar joint instability more, the ITCL or the CFL, cannot be ascertained with the experimental setup of this study. It would have been necessary to have two groups, one cutting the ITCL first and the other cutting the CFL first, requiring twice the number of cadaveric specimens. It would certainly be interesting to evaluate results of a similar experimental protocol sectioning the CFL first instead of the ITCL.

Relatively few studies describe surgical techniques for treatment of subtalar instability. Though the trend is towards anatomic reconstruction of the damaged structures and use of arthroscopically assisted techniques, most studies are limited to retrospective case series with a limited number of patients and variable follow-up, without any biomechanical rationale supporting the proposed techniques [11, 14, 21]. Several authors proposed anatomic reconstruction of the inter-talocalcaneal ligament, considered by many the most potent ligament and main stabiliser of the subtalar joint [6, 15, 18, 21, 34, 40]. The surgical technique described has many advantages compared to other techniques, such as (1) anatomic tunnel placement under direct arthroscopic visualisation, without the need of fluoroscopy; (2) the creation of a talar half tunnel avoids the complications associated with drilling a complete tunnel, such as talar neck fracture and injury to the anterior tendinous and neurovascular structures; (3) cortical fixation on the anterior talar surface allows for a more stable fixation and reduces the need for immobilisation; (4) the use of allograft avoids donor site morbidity and (5) simplicity of this technique allows for a relatively brief learning curve and a short duration of surgery, of particular interest in cases of combined tibiotalar and subtalar instability that also require reconstruction of the lateral ankle ligaments.

Several strengths can be found in this study: no evaluations of angular stability through biomechanical studies after reconstructing the ITCL are known to us. It is a cadaveric study with a relatively large sample size and a rigorous protocol to minimise measurement errors using a validated measurement device already used in other biomechanical studies of the ankle [7].

We are aware of the limitations of our study. First, our analysis has the limitations inherent to cadaver studies. Allograft reconstruction was studied without considering the fibrosis of the subtalar joint that would be present in a live patient with injured ligaments and that would also contribute to joint stability. The effect of the posterior tibialis, flexor digitorum communis and flexor hallucis longus tendons, which cross the sustentaculum tali inferiorly and contribute to dynamic stability of the hindfoot, could not be assessed in our study, which was limited to the intrinsic stability provided by osseous and ligamentous structures. Rather than a flaw, this could be seen as a validation of the study’s original goal, which was to assess the effect of ligament injury and whether reconstruction returned stability to near normal values, regardless of the role of dynamic stabilisers that would need to compensate for insufficient ligamentous stability. Another criticism to our study is that each cadaveric specimen is also the control of each technique. Using a separate control group would have increased the number of specimens needed. It was not possible to use pairs of ankles proceeding from the same cadaver, in which the technique could have been performed on one side with the other acting as a control. This disadvantage was tried to be minimised in this study using a logical sequence, analysing the intact joint as a control first, then progressively destabilising the joint and finally observing the effect of ligament reconstruction, before sectioning the extrinsic stabiliser of the subtalar joint.

Second, the limitations inherent to the measurement technique must be mentioned. The force applied manually to perform the different manoeuvres was not measured. These manoeuvres are actually a dynamic measurement, and realistic reproduction of the clinical examination of instability would have been impossible it tensiometers had been used to control the forces used. Variability was tried to be reduced by having all the manoeuvres, repeated three times for each test, performed by single researcher, an orthopaedic surgeon with ample clinical experience in this area. The fact that the dynamic stabilisers are not reproduced must be added to the limitations of biomechanical cadaver studies.

When progressively sectioning ligamentous stabilisers, significant values of angular displacement were measured with the inversion and eversion stress tests of the subtalar joint, two manoeuvres used in clinical practice but not evaluated to this level of detail, making this study clinically relevant. The ITCL was found to significantly add to subtalar joint laxity, and that joint kinematics against inversion were restored by allograft reconstruction of the ITCL, even in cases with a combined injury of the CL and ITCL.

## Conclusion

The ITCL was found to be an important stabiliser of the subtalar joint in the coronal and sagittal planes, in inversion as well as in eversion.

The IER and the CL were stabilisers in the axial plane against inversion and eversion, respectively. Sectioning the CFL added to instability against eversion in the axial and coronal planes, and against inversion in the coronal plane.

Reconstructing the ITCL using tendon graft significantly stabilised the subtalar joint in the axial and sagittal planes against eversion and in the axial and coronal planes against inversion, immediately after surgery.

## References

[1] Aynardi M, Pedowitz DI, Raikin SM (2015). Subtalar instability. Foot Ankle Clin.

[2] Bell KL, King BW, Sangeorzan BJ (2023). Acute and chronic subtalar joint instability: does it really exist?. Foot Ankle Clin.

[3] Burssens A, Krähenbühl N, Lenz AL, Howell K, Zhang C, Sripanich Y, Saltzman CL, Barg A (2022). Interaction of loading and ligament injuries in subtalar joint instability quantified by 3D weightbearing computed tomography. J Orthop Res.

[4] Choisne J, Ringleb SI, Samaan MA, Bawab SY, Naik D, Anderson CD (2012). Influence of kinematic analysis methods on detecting ankle and subtalar joint instability. J Biomech.

[5] D’Hooghe P, Pereira H, Kelley J, Anderson N, Fuld R, Kumparatana P, Baldini T, Hunt KJ (2020). The CFL fails before the ATFL immediately after combined ligament repair in a biomechanical cadaveric model. Knee Surg Sports Traumatol Arthrosc.

[6] Gomes TM, Oliva XM, Viridiana Sanchez E, Soares S, Diaz T (2023). Anatomy of the ankle and subtalar joint ligaments: what we do not know about it?. Foot Ankle Clin.

[7] Guerra-Pinto F, Cunha J, Sousa L, Gomes TM, Andrade R, Oliva XM, Consciência JG, Fernandes PR (2020). Gravity stress tibiotalar laxity evaluation with a biomedical gyroscopes device - cadaver study with progressive sectioning of lateral ankle ligaments. J Exp Orthop.

[8] Heilman AE, Braly WG, Bishop JO, Noble PC, Tullos HS (1990). An anatomic study of subtalar instability. Foot Ankle.

[9] Iglesias-Durán E, Guerra-Pinto F, García-Esteo F, Vilá-Rico J (2020). Anatomic arthroscopic graft reconstruction of the interosseous talocalcaneal ligament for subtalar instability. Arthrosc Tech.

[10] Jotoku T, Kinoshita M, Okuda R, Abe M (2006). Anatomy of ligamentous structures in the tarsal sinus and canal. Foot Ankle Int.

[11] Jung H-G, Kim T-H (2012). Subtalar instability reconstruction with an allograft: technical note. Foot Ankle Int.

[12] Kamiya T, Kura H, Suzuki D, Uchiyama E, Fujimiya M, Yamashita T (2009). Mechanical stability of the subtalar joint after lateral ligament sectioning and ankle brace application: a biomechanical experimental study. Am J Sports Med.

[13] Karlsson J, Eriksson BI, Renström PA (1997). Subtalar ankle instability. A review. Sports Med.

[14] Karlsson J, Eriksson BI, Renström P (1998). Subtalar instability of the foot. A review and results after surgical treatment. Scand J Med Sci Sports.

[15] Kato T (1995). The diagnosis and treatment of instability of the subtalar joint. J Bone Joint Surg Br.

[16] Keefe DT, Haddad SL (2002). Subtalar instability. Etiology, diagnosis, and management. Foot Ankle Clin.

[17] Kim J, Kim GL, Kim T, Cho J (2023). Evaluation of chronic ankle instability and subtalar instability using the angle between the anterior talofibular ligament and calcaneofibular ligament. Knee Surg Sports Traumatol Arthrosc.

[18] Kjaersgaard-Andersen P, Wethelund JO, Helmig P, Søballe K (1988). The stabilizing effect of the ligamentous structures in the sinus and canalis tarsi on movements in the hindfoot. An experimental study. Am J Sports Med.

[19] Knudson GA, Kitaoka HB, Lu CL, Luo ZP, An KN (1997). Subtalar joint stability. Talocalcaneal interosseous ligament function studied in cadaver specimens. Acta Orthop Scand.

[20] Li S-Y, Hou Z-D, Zhang P, Li H-L, Ding Z-H, Liu Y-J (2013). Ligament structures in the tarsal sinus and canal. Foot Ankle Int.

[21] Liu C, Jiao C, Hu Y, Guo QW, Wand C, Ao Y (2011). Interosseous talocalcaneal ligament reconstruction with hamstring autograft under subtalar arthroscopy: case report. Foot Ankle Int.

[22] Martin LP, Wayne JS, Monahan TJ, Adelaar RS (1998). Elongation behavior of calcaneofibular and cervical ligaments during inversion loads applied in an open kinetic chain. Foot Ankle Int.

[23] Michels F, Pereira H, Calder J, Matricali G, Glazebrook M, Guillo S, Karlsson J, Acevedo J, Batista J, Bauer T, Calder J, Carreira D, Choi W, Corte-Real N, Glazebrook M, Ghorbani A, Giza E, Guillo S, Hunt K, Karlsson J, Kong SW, Lee JW, Michels F, Molloy A, Mangone P, Matsui K, Nery C, Ozeki S, Pearce C, Pereira H, Perera A, Pijnenburg B, Raduan F, Stone J, Takao M, Tourné Y, Vega J, ESSKA-AFAS Ankle Instability Group (2018). Searching for consensus in the approach to patients with chronic lateral ankle instability: ask the expert. Knee Surg Sports Traumatol Arthrosc.

[24] Michels F, Clockaerts S, Van Der Bauwhede J, Stockmans F, Matricali G (2020). Does subtalar instability really exist? A systematic review. Foot Ankle Surg.

[25] Michels F, Matricali G, Vereecke E, Dewilde M, Vanrietvelde F, Stockmans F (2021). The intrinsic subtalar ligaments have a consistent presence, location and morphology. Foot Ankle Surg.

[26] Michels F, Stockmans F, Pottel H, Matricali G (2022). Reconstruction of the cervical ligament in patients with chronic subtalar instability. Foot Ankle Surg.

[27] Michels F, Taylan O, Stockmans F, Vereecke E, Scheys L, Matricali G (2022). The different subtalar ligaments show significant differences in their mechanical properties. Foot Ankle Surg.

[28] Michels F, Vereecke E, Matricali G (2023). Role of the intrinsic subtalar ligaments in subtalar instability and consequences for clinical practice. Front Bioeng Biotechnol.

[29] Michelson J, Hamel A, Buczek F, Sharkey N (2004). The effect of ankle injury on subtalar motion. Foot Ankle Int.

[30] Mittlmeier T, Rammelt S (2018). Update on Subtalar Joint Instability. Foot Ankle Clin.

[31] Pellegrini MJ, Glisson RR, Wurm M, Ousema PH, Romash MM, Nunley JA, Easley ME (2016). Systematic Quantification of Stabilizing Effects of Subtalar Joint Soft-Tissue Constraints in a Novel Cadaveric Model. J Bone Joint Surg Am.

[32] Pereira BS, Andrade R, Espregueira-Mendes J, Marano RPC, Oliva XM, Karlsson J (2021). Current Concepts on Subtalar Instability. Orthop J Sports Med.

[33] Pisani G, Pisani PC, Parino E (2005). Sinus tarsi syndrome and subtalar joint instability. Clin Podiatr Med Surg.

[34] Ringleb SI, Dhakal A, Anderson CD, Bawab S, Paranjape R (2011). Effects of lateral ligament sectioning on the stability of the ankle and subtalar joint. J Orthop Res.

[35] Sangeorzan A, Sangeorzan B (2018). Subtalar Joint Biomechanics: From Normal to Pathologic. Foot Ankle Clin.

[36] Song WT, Lee J, Lee JH, Lim J-W, Im J-M, Lee D-O, Jung H-G (2021). A high rate of talocalcaneal interosseous ligament tears was found in chronic lateral ankle instability with sinus tarsi pain. Knee Surg Sports Traumatol Arthrosc.

[37] Tan CY, Thevendran G (2023). Subtalar instability. J Orthop Surg Hong Kong.

[38] Thermann H, Zwipp H, Tscherne H (1997). Treatment algorithm of chronic ankle and subtalar instability. Foot Ankle Int.

[39] Tochigi Y, Takahashi K, Yamagata M, Tamaki T (2000). Influence of the interosseous talocalcaneal ligament injury on stability of the ankle-subtalar joint complex–a cadaveric experimental study. Foot Ankle Int.

[40] Tochigi Y, Amendola A, Rudert MJ, Baer TE, Brown TD, Hillis SL, Saltzman CL (2004). The role of the interosseous talocalcaneal ligament in subtalar joint stability. Foot Ankle Int.

[41] Weindel S, Schmidt R, Rammelt S, Claes L, von Campe A, Rein S (2010). Subtalar instability: a biomechanical cadaver study. Arch Orthop Trauma Surg.

